# A Cyclin-Dependent Kinase that Promotes Cytokinesis through Modulating Phosphorylation of the Carboxy Terminal Domain of the RNA Pol II Rpb1p Sub-Unit

**DOI:** 10.1371/journal.pone.0000433

**Published:** 2007-05-09

**Authors:** Jim Karagiannis, Mohan K. Balasubramanian

**Affiliations:** 1 Laboratory of Cell Division, Temasek Life Sciences Laboratory, Singapore, Singapore; 2 Department of Biological Sciences, National University of Singapore, Singapore, Singapore; Northwestern University, United States of America

## Abstract

In *Schizosaccharomyces pombe,* the nuclear-localized kinase, Lsk1p, promotes cytokinesis by positively regulating the Septation Initiation Network (SIN). Although a member of the cyclin-dependent kinase (CDK) family, neither a cyclin partner nor a physiological target has been identified. In this report we identify a cyclin, Lsc1p, that physically interacts and co-localizes with Lsk1p. Furthermore, *lsk1*Δ, *lsc1*Δ, as well as kinase-dead *lsk1-K306R* mutants, display highly similar cytokinesis defects. Lsk1p is related to CDKs that phosphorylate the carboxy-terminal domain (CTD) of the largest sub-unit of RNA polymerase II (Rpb1p). Interestingly, we find that Lsk1p and Lsc1p are required for phosphorylation of Ser-2 residues found in the heptad repeats of the CTD. To determine if Rpb1p could be a physiological target, we replaced the native *rpb1* gene with a synthetic gene encoding a Rpb1p protein in which Ser-2 was substituted with the non-phosphorylatable amino-acid alanine in all heptads. Cells carrying this allele were similar to *lsk1*Δ mutants: They were viable, displayed genetic interactions with the SIN, and were unable to complete cytokinesis upon perturbation of the cell division machinery. We conclude that Ser-2 phosphorylation of the CTD heptads plays a novel physiological role in the regulation of cytokinesis.

## Introduction

In *Schizosaccharomyces pombe* cytokinesis is monitored by a checkpoint system that scrutinizes the integrity of the actomyosin ring. Upon perturbation of the cell division machinery–either by the addition of drugs, or the introduction of temperature sensitive mutations in the cytokinetic apparatus–the checkpoint is able to delay progression into the subsequent mitosis, as well as promote actomyosin ring integrity, re-assembly, and constriction [1]–[5]. Critical regulators of the checkpoint include the Septation Initiation Network (SIN) and the Cdc14p family phosphatase, Clp1p/Flp1p.

The SIN defines a network of essential genes that are required for the constriction, but not the assembly, of the actomyosin ring [6]–[8]. In contrast, *clp1* encodes a non-essential phosphatase whose loss confers only weak cytokinesis defects during typical growth [1], [9]. However, under conditions in which the cytokinesis machinery is partially compromised, *clp1*Δ cells display a lethal cytokinesis phenotype due to their inability to prolong the duration of SIN signaling [2], [3].

In addition to the SIN and Clp1p, the nuclear-localized kinase, Lsk1p, also plays an important role in the checkpoint response. Similar to *clp1*Δ cells, *lsk1*Δ mutants appear normal under typical growth conditions, but display striking cytokinesis defects upon perturbation of the actomyosin ring. Not surprisingly, as demonstrated by synthetic lethal genetic interactions between *lsk1*Δ and hypo-active SIN mutants, as well as *lsk1*Δ mediated suppression of SIN hyper-activation, Lsk1p functions as a positive regulator of the SIN [10].

Lsk1p displays significant homology to a sub-group of CDKs that phosphorylate a unique carboxy-terminal domain (CTD) of the largest sub-unit of RNA polymerase II (Rpb1p). This domain is a highly conserved component of the RNA pol II complex and is composed of a variable number of repeats (between 20 and 50 depending on species) of the seven amino acid sequence YSPTSPS. This sequence can be phosphorylated on both Ser-2 and Ser-5 residues by a sub-family of CDKs that are related to, but distinct from traditional CDKs involved in cell cycle control. The phosphorylation status of the CTD influences initiation and elongation by the pol II complex, as well as the binding of a wide variety of accessory factors required for the proper splicing, capping, and poly-adenylation of transcripts [11]–[14].

Lsk1p, as well as *S. pombe* Cdk9p, display significant sequence similarity to human Cdk9p. Human Cdk9p, together with cyclin T, forms the P-TEFb complex. This complex targets Ser-2 residues of the CTD and promotes productive transcript elongation subsequent to its recruitment to the RNA pol II complex [15]. In addition to human Cdk9p, Lsk1p shows significant sequence similarity to *S. cerevisiae* Bur1p and Ctk1p (the closest relative of Lsk1p in *S. cerevisiae*). Both Bur1p and Ctk1p (a Ser-2 CTD kinase) play general roles in promoting transcript elongation that may be related to the modulation of histone H3 K4 methylation [16]–[21]. *S. cerevisiae* Ctk1p plays a specific role in the regulation of the DNA damage response in budding yeast [22], [23].

In this report we identify the cyclin partner of Lsk1p, and show that the Lsk1p-Lsc1p complex is required for Ser-2 phosphorylation of the CTD of Rpb1p. We show that Rpb1p is likely the physiologically relevant target of the Lsk1p-Lsc1p complex in terms of its role in the cytokinesis checkpoint. We also demonstrate that over-expression of the CTD phosphatase, Fcp1p, as well as mutations in Rpb1p that substitute alanine for serine in the CTD result in cytokinesis defects that are characteristic of *lsk1*Δ mutants. These data support a role for Ser-2 phosphorylation of the heptad repeats of Rpb1p in the regulation of cytokinesis.

## Results

### Deletion mutants of the cyclin, *lsc1*, display cytokinesis defects similar to those displayed by *lsk1*Δ strains

**Figure 1:**
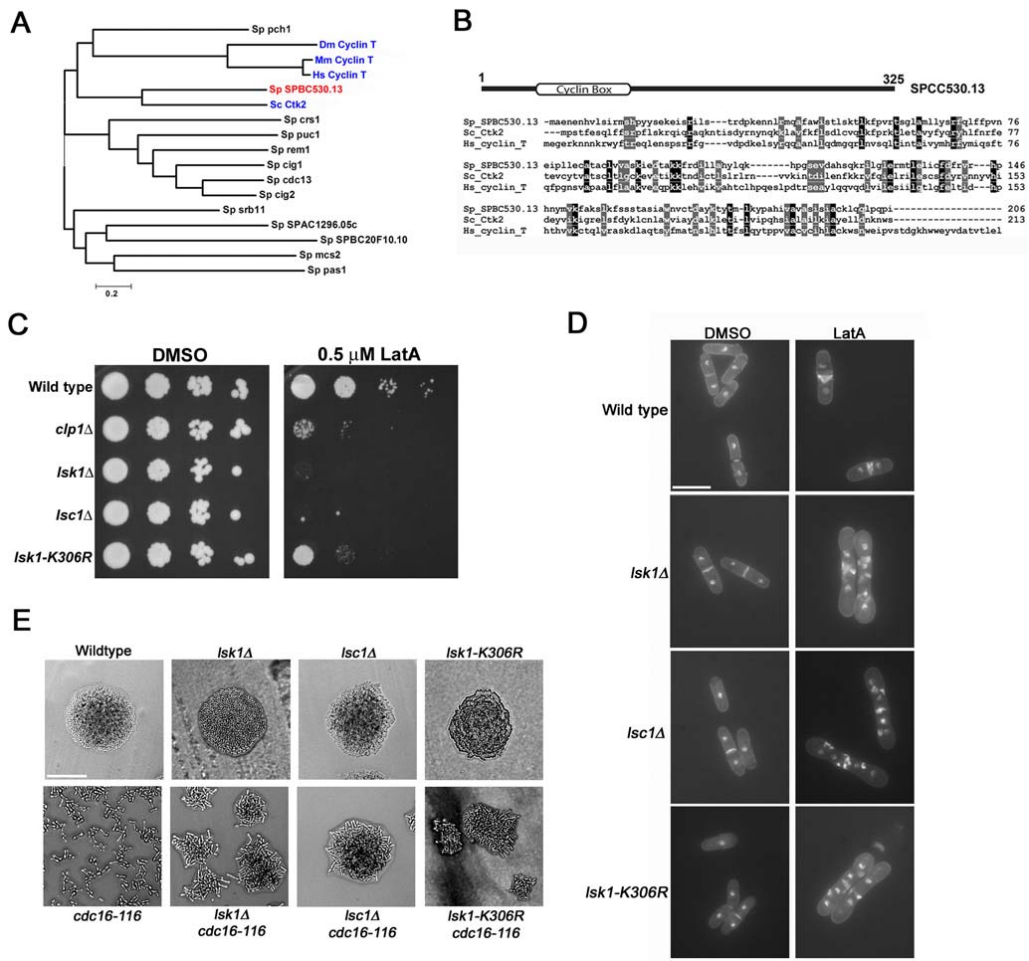
Strains bearing the *lsc1Δ* or *lsk1-K306R* mutations display highly similar cytokinesis defects as compared to *lsk1Δ* mutants. (A) A phylogeny analyzing the relationship between *S. pombe* cyclins (black type) and the cyclin partners of Lsk1p relatives in budding yeast, mouse, human, and *Drosophila* (blue type). The most likely cyclin partner of Lsk1p based on this analysis, SPCC530.13, is shown in red. The phylogeny was created by MEGA version 3.1 software using the neighbor-joining algorithm. Sp, *Schizosacharomyces pombe*; Dm, *Drosophila melanogaster*; Hs *Homo sapiens*; Mm *Mus musculus*; Sc *Saccharomyces cerevisiae*. (B) ClustalW alignment comparing *S. pombe* SPCC530.13, *S. cerevisiae* Ctk2p, and human cyclin T. (C) Ten-fold serial dilutions of logarithmically growing cultures were plated onto YES plates containing 0.5 µM LatA or DMSO (solvent control) and incubated at 32°C for 3 days. (D) Cells of the indicated genotype were grown to mid-log phase at 32°C and then treated with 0.3 µM LatA for 5 hours before being fixed and stained with DAPI (nuclei) and aniline blue (cell wall/septa). Bar, 10 µM. (E) Cells of the indicated genotype were freshly streaked to YES plates and incubated for 24 hours at 36°C. Bar, 50 µM.

Cyclin dependent kinases are positively regulated via their physical interaction with cyclins [24]. Since the identification of a cyclin partner would allow us to assess whether Lsk1p activity was regulated by modifications in cyclin stability and/or localization we decided to determine whether such a physiologically relevant cyclin sub-unit existed in *S. pombe*. To this end we compared the sequence of all 13 cyclins in the *S. pombe* genome to the cyclin partners of Lsk1p relatives in budding yeast, mouse, human, and *Drosophila*. A phylogeny created by MEGA version 3.1 software using the neighbor-joining algorithm [25] predicted that the open reading frame designated SPBC530.13 was the best candidate (Figure 1A). SPBC530.13 encodes a previously uncharacterized protein of 325 amino acids and contains one copy of the conserved cyclin box in its N-terminus. Homology to yeast and human counterparts is mainly in the N-terminal half of the protein containing this domain. Its C-terminus bears no other motifs to suggest other potential biochemical functions (Figure 1B). 

To further explore the possibility that SPBC530.13 was indeed the cyclin partner of Lsk1p, we deleted the ORF and examined the loss of function phenotype. We began by assaying the sensitivity of the deletion strain to low doses of the actin polymerization-inhibitor, Latrunculin A. As previously demonstrated, treatment with LatA in the range of 0.2–0.5 µM leads to a Clp1p dependent delay in mitotic entry and the extended activation of the SIN. This leads to a prolonged cytokinesis-competent state characterized by continuous repair and re-establishment of the actomyosin ring. Thus, such treatment can be employed as a tool to activate the cytokinesis checkpoint [2], [3], [10].

When mutants deleted for SPBC530.13 were compared to *clp1*Δ and *lsk1*Δ deletions, they displayed a similar hyper-sensitivity to LatA (Figure 1C). In addition, deletion of *lsc1* gave rise to phenotypes characteristic of *lsk1*Δ mutants. These included the inability to complete septum formation upon LatA treatment (Figure 1D; Table 1), as well as the ability to suppress the lethal, multi-septate phenotype conferred by the constitutive hyperactivation of the SIN (as assayed using the temperature sensitive *cdc16-116* mutation) [26], [27] (Figure 1E). Thus SPCC530.13 will be referred to hereafter as *lsc1* (latrunculin sensitive cyclin knockout).

**Table 1:** Mean percentage of cells (±standard deviation) displaying the indicated phenotype after 5 hours treatment with 0.3 µM Latrunculin A (n = 3).

Genotype	Uni-nucleate	Bi-Nucleate (Complete Septum)	Bi-Nucleate (Fragmented Septum)	Tetra-Nucleate (Fragmented Septum)	>4 Nuclei
Wild type	10±1	71±7	17±6	3±2	0
*lsk1Δ*	2±1	9±4	39±5	49±6	2±1
*lsc1Δ*	2±1	13±4	41±2	43±6	2±1
*lsk1-K306R*	7±2	21±2	35±7	35±4	2±1
*rpb1-12xCTD*	12±2	64±4	19±5	4±1	0
*rpb1-12xS2ACTD*	7±2	20±2	42±3	30±3	1±1

Lastly, to provide more direct evidence that the kinase activity of the putative Lsk1p-Lsc1p complex was indeed physiologically relevant, as opposed to some other property of the complex, we created a kinase dead mutant of Lsk1p by mutating the conserved lysine-306 residue to arginine. *lsk1-K306R, lsk1*Δ, and *lsc1*Δ mutants behaved similarly with respect to LatA sensitivity, and genetic interactions with the SIN (Figure 1, C–E; Table 1).

### Lsk1p and Lsc1p co-localize to the nucleus and form a physical complex in vivo

If Lsk1p and Lsc1p were indeed part of a functional CDK complex then they should co-localize to the nucleus, and physically interact in vivo. In addition, mutations in either the kinase or cyclin sub-unit might affect localization and interaction with the associating partner (e.g. deletion of the B-type cyclin, *cdc13* in *S. pombe* prevents the nuclear accumulation of the kinase subunit, Cdc2p) [28]. To first determine the localization of Lsc1p, and also to determine whether modulation of cyclin localization might play a regulatory role in controlling the activity of the constitutively nuclear, Lsk1p, we examined the localization of Lsc1-GFP fusion proteins expressed from a construct integrated at the *lsc1* locus and under the control of the native *lsc1* promoter (see Materials and Methods). Similarly to Lsk1p-GFP, Lsc1p-GFP was constitutively localized to the nucleus. Its localization was not affected by changes in temperature, cell cycle position, or by treatment with LatA (data not shown).

**Figure 2:**
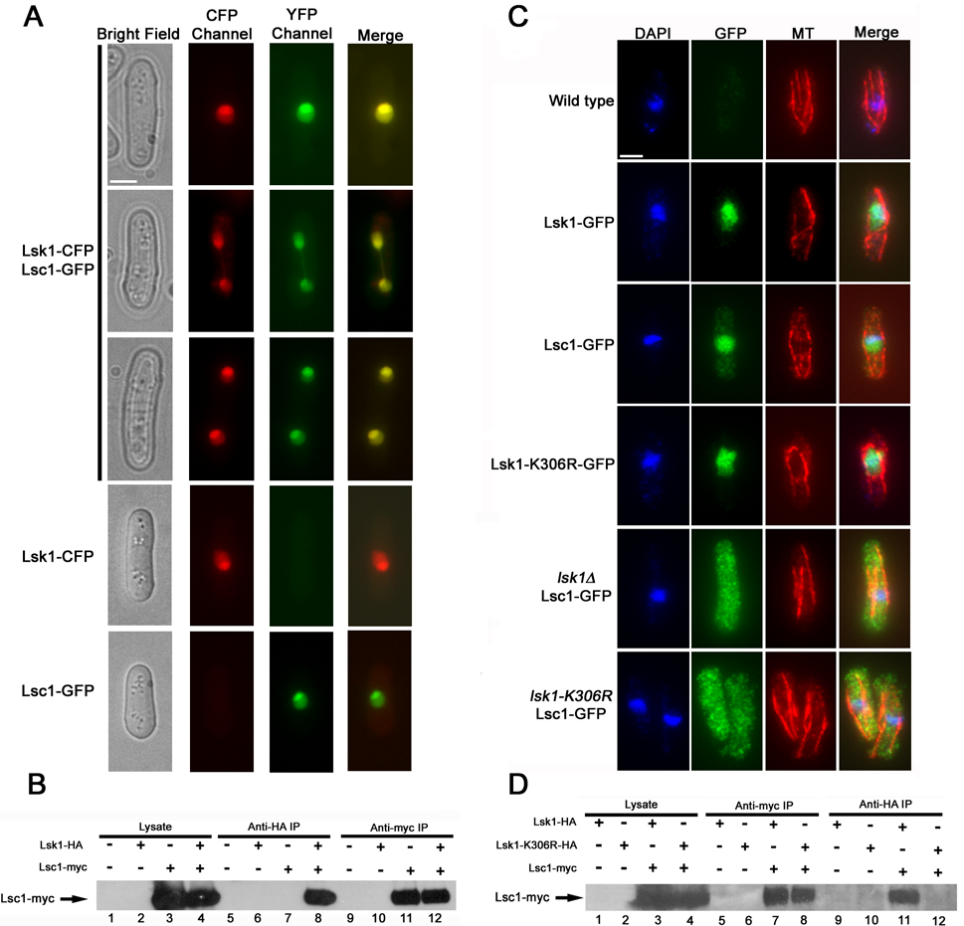
Lsk1p and Lsc1p co-localize to the nucleus and physically interact in a manner dependent on Lsk1p kinase activity. (A) Cells expressing Lsk1-CFP or Lsc1-GFP under the control of the thiamine repressible *nmt1* promoter were grown for 12 hours in minimal media in the absence of thiamine, and then imaged in the CFP and YFP channels, respectively. Bar, 3 µM. (B) Wild-type, Lsk1-HA, Lsc1-myc, and Lsk1-HA Lsc1-myc cells were grown to mid-log phase and lysed under native conditions. A portion of the lysates were subjected to anti-HA and anti-myc immunoprecipitations. Both total lysates and immunoprecipitates were resolved by SDS-PAGE and immunoblotted with antibodies specific for the myc epitope. (C) Cells of the indicated genotype, carrying integrated GFP tagged versions of Lsk1p or Lsc1p under the control of their native promoter, were grown to mid-log phase in YES media, fixed, and then stained with DAPI (nuclei) and antibodies specific for microtubules and GFP. Bar, 3 µM. (D) Lsk1-HA, Lsk1-K306R-HA, Lsk1-HA Lsc1-myc, and Lsk1-K306R-HA Lsc1-myc cells were grown to mid-log phase in YES and lysed under native conditions. A portion of the lysates were subjected to anti-HA and anti-myc immunoprecipitations. Both total lysates and immunoprecipitates were resolved by SDS-PAGE and immunoblotted with antibodies specific for the myc epitope.

To assay co-localization we expressed Lsk1-CFP and Lsc1-GFP fusion proteins under the control of the thiamine repressible *nmt1* promoter, and subsequently expressed them in wild-type fission yeast cells. Consistent with a model in which Lsk1p and Lsc1p formed a complex, both the kinase and cyclin co-localized to the nucleus (Figure 2A). Furthermore, co-immunoprecipitation experiments showed that Lsc1-myc fusion proteins could be detected in anti-HA immunoprecipitates in strains expressing Lsk1-HA, but not in similar immunoprecipitates from strains expressing non-HA tagged Lsk1p (Figure 2B). Interestingly, when Lsc1p localization was monitored in *lsk1Δ* or in *lsk1-K306R* mutants, Lsc1p was unable to enter the nucleus and was found distributed throughout the cytoplasm (Figure 2C). Importantly, GFP-tagged *lsk1-K306R* proteins displayed a similar localization to that seen by wild-type Lsk1p, indicating that Lsc1p mis-localization was not simply an indirect consequence of mis-directed *lsk1-K306R* encoded protein. Lsk1p localization was not affected by deletion of *lsc1* (data not shown).

As expected HA-tagged *lsk1-K306R* proteins, unlike wild-type Lsk1-HA proteins, failed to show any physical interaction with Lsc1p (Figure 2D). Thus, Lsk1p kinase activity is required for both Lsc1p nuclear localization as well as the assembly of a functional Lsk1p-Lsc1p complex. Taking all data together, our results strongly indicate that Lsk1p and Lsc1p form a bona fide CDK-cyclin complex that plays an important role in the completion of cytokinesis through its actions as a positive regulator of the SIN. However, no physiological target of the complex was known.

### Lsk1p and Lsc1p are required for Ser-2 phosphorylation of the CTD of Rpb1p

CDKs can be broadly divided into two groups: those that are involved in regulating the control of cell cycle progression, and those that play a role in transcriptional control by directly phosphorylating the RNA polymerase II machinery [11]. One specific sub-group is known to phosphorylate the CTD of the largest sub-unit of RNA polymerase II. Since the Lsk1p-Lsc1p CDK was most closely related to this latter sub-groups of CDKs we considered the possibility that it too might affect CTD phosphorylation status.

**Figure 3:**
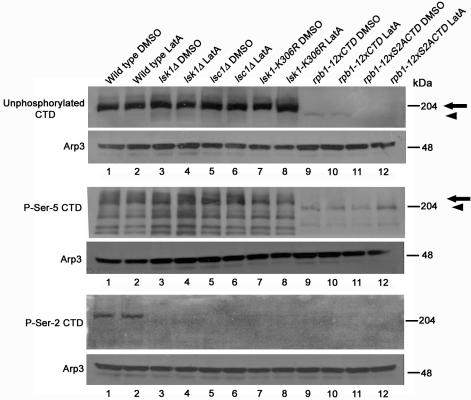
Lsk1p and Lsc1p are required for Ser-2 phosphorylation of the heptad repeats found in the carboxy-terminal domain of the Rpb1p sub-unit of RNA pol II. Cells were grown to mid-log phase in YES, and then treated either with 0.3 µM LatA or DMSO (solvent control) for 2 hours. Total lysates were resolved by SDS-PAGE and immunoblotted with antibodies specific for the unphosphorylated form of the CTD (8WG16), the Ser-5 phosphorylated form of the CTD (H14), the Ser-2 phosphorylated form of the CTD (H5), as well as antibodies specific for the Arp3 protein (loading control). Arrowheads indicate bands of increased mobility derived from *rpb1-12xCTD* and *rpb1-12xS2ACTD* mutants, while arrows indicate Rpb1p protein bands derived from the wild-type *rpb1* locus.

We assayed this possibility by utilizing phosphospecific antibodies against the Ser-2 and Ser-5 phosphorylated forms of the CTD. When protein extracts were analyzed, Ser-2 phosphorylated CTD could be detected in wild-type strains, but not in *lsk1*Δ, *lsc1*Δ, or *lsk1-K306R* mutants (Figure 3, bottom panel, lanes 1–8). Equal loading was confirmed by blotting for the Arp3p protein. In contrast Ser-5 phosphorylated CTD, as well as the level of unphosphorylated CTD, was not affected in these mutants (Figure 3, top and middle panels, lanes 1–8). Experiments assaying the effect of LatA treatment on phospho-CTD levels also did not reveal any differences between treated and non-treated cells in any of these strains (Figure 3).

### Over-expression of the CTD phosphatase Fcp1p results in cytokinesis defects

Given that the Lsk1p-Lsc1p complex was required for Ser-2 phosphorylation of the CTD, we next considered the possibility that a reduction in Ser-2 phosphorylation was indeed responsible for the observed cytokinetic defects in *lsk1*Δ and *lsc1*Δ mutants. Two alternate experimental approaches were chosen to test this hypothesis. In the first method, we examined the effects of over-expressing the Fcp1p phosphatase under the control of the moderate strength thiamine repressible *nmt41* promoter. Fcp1p is a *S. pombe* phosphatase that has been shown to preferentially de-phosphorylate synthetic CTD peptides phosphorylated on Ser-2 residues as opposed to similar peptides phosphorylated on Ser-5 [29], [30].

Since the localization of Fcp1p had not been experimentally determined, and since it would need to localize to the nucleus in order to be a physiologically relevant Ser-2 CTD phosphatase, we first examined its localization by creating a C-terminally tagged GFP fusion. Similar to Lsk1p, and Lsc1p, Fcp1p displayed a constitutive nuclear localization that was not affected by cell cycle position, temperature, or the presence of LatA (Figure S1; data not shown).

**Figure 4:**
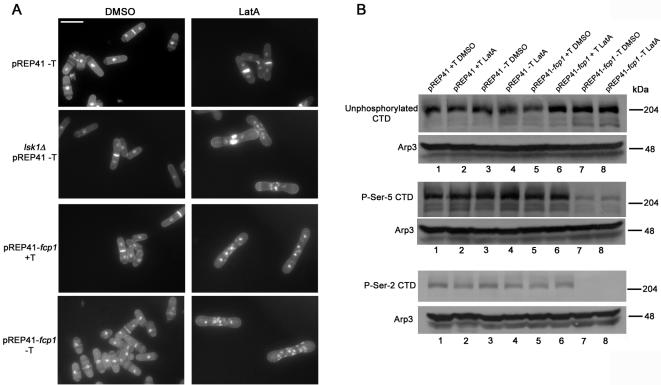
Over-expression of the CTD phosphatase, Fcp1p, causes defects in cytokinesis. (A) Cells of the indicated genotype were grown to mid-log phase in minimal media containing thiamine and then re-cultured in the presence or absence of thiamine for a further 18 hours. Cells were then treated with 0.3 µM LatA or DMSO (solvent control) for 5 hours and subsequently fixed and stained with DAPI (nuclei) and aniline blue (cell wall/septa). Bar, 10 µM. (B) Cells of the indicated genotype were grown to mid-log phase in minimal media containing thiamine and then re-cultured in the presence or absence of thiamine for a further 18 hours. Cells were then treated with 0.3 µM LatA or DMSO (solvent control) for a further 2 hours. Total lysates were resolved by SDS-PAGE and immunoblotted with antibodies specific for the unphosphorylated form of the CTD (8WG16), the Ser-5 phosphorylated form of the CTD (H14), the Ser-2 phosphorylated form of the CTD (H5), as well as antibodies specific for the Arp3 protein (loading control).

We next examined the phenotypic consequences of Fcp1p over-expression. Remarkably, when Fcp1p over-expressing cells were treated with LatA, they displayed a characteristic inability to complete cytokinesis in both repressing and non-repressing conditions. This was in contrast to cells carrying vector alone, which were able to complete septum formation upon similar treatment (Figure 4A; Table 2). The fact that Fcp1p over-expressing cells behaved similarly in both the presence and absence of thiamine was somewhat surprising. However, this likely reflects the fact that repression of the *nmt41* promoter is incomplete even in the presence of excess repressor [31]. Therefore, this suggests that only slight over-expression of Fcp1p is required to bring about a physiological effect.

**Table 2:** Mean percentage of cells (±standard deviation) displaying the indicated phenotype after 5 hours treatment with 0.3 µM Latrunculin A (n = 3).

Genotype	Vector	Induced/Repressed	Uni-nucleate	Bi-Nucleate (Complete Septum)	Bi-Nucleate (Fragmented Septum)	Tetra-Nucleate (Fragmented Septum)	>4 Nuclei
Wild type	pREP41	Induced	25±3	63±4	11±3	1±1	0
*lsk1Δ*	pREP41	Induced	29±6	13±5	27±4	29±4	4±2
Wild type	pREP41-fcp1	Repressed	24±5	15±5	31±6	26±7	5±2
Wild type	pREP41-fcp1	Induced	21±6	19±3	27±3	30±4	4±3

We next examined the effects of Fcp1p over-expression on the levels of Ser-2 and Ser-5 phosphorylated CTD. When extracts of cells expressing Fcp1p under repressing conditions (plus thiamine) were examined, Ser-5 phosphorylated CTD could be readily detected. However, under non-repressing conditions (minus thiamine) the levels of Ser-5 phosphorylated CTD were consistently reduced compared to repressed controls (Figure 4B, middle panel, lanes 5–8). In contrast, Ser-5 phosphorylated CTD could be detected at similar levels in the presence or absence of thiamine in cells expressing empty vector (Figure 4B, middle panel, lanes 1–4). Furthermore, the levels of unphosphorylated CTD were unaffected by Fcp1p over-expression (Figure 4B, top panel).

Since, Fcp1p over-expressing cells displayed a strong cytokinesis phenotype in the presence of thiamine, we expected to observe a reduction of phospho-Ser-2 CTD under both repressing and non-repressing conditions. However, while Ser-2 phosphorylated CTD could not be detected in the absence of thiamine, it was clearly observed in cells grown in the presence of the repressor (Figure 4B, bottom panel, lanes 5–8). These results could suggest that slight changes in Ser-2 phosphorylation, beyond the resolution of detection of our Western blots, may be sufficient to result in a physiological effect. Alternatively, the dynamic turn-over of phosphate groups, as opposed to the average level of phosphorylation, may be the crucial factor in terms of phenotypic consequence. As with the level of Ser-5 phosphorylated CTD, the level of Ser-2 phosphorylated CTD was not altered when empty vector controls were assayed in repressing or non-repressing conditions (Figure 4B, bottom panel, lanes 1–4). In addition, LatA treatment had no effect on non-phosphorylated, or phospho-CTD levels in either repressing or non-repressing conditions (Figure 4B).

Although these experiments were suggestive of a role for CTD phosphorylation in the control of cytokinesis, they suffered from two limitations. First, since Fcp1p was being over-expressed, it was conceivable that it might be promiscuously de-phosphorylating substrates other than the CTD. Secondly, Fcp1p over-expression was affecting both phospho-Ser-2 and phospho-Ser-5 CTD levels. Thus it was not possible to determine whether changes in phospho Ser-5 and/or phospho Ser-2 levels were the primary cause of the observed defects in cytokinesis. We thus sought more direct evidence by creating an Rpb1p mutant in which the serine-2 residues of the CTD heptads were mutated to alanine to mimic the non-phosphorylated state.

### Lsk1p and Lsc1p facilitate the successful completion of cytokinesis by promoting Ser-2 phosphorylation of the CTD of Rpb1p

**Figure 5:**
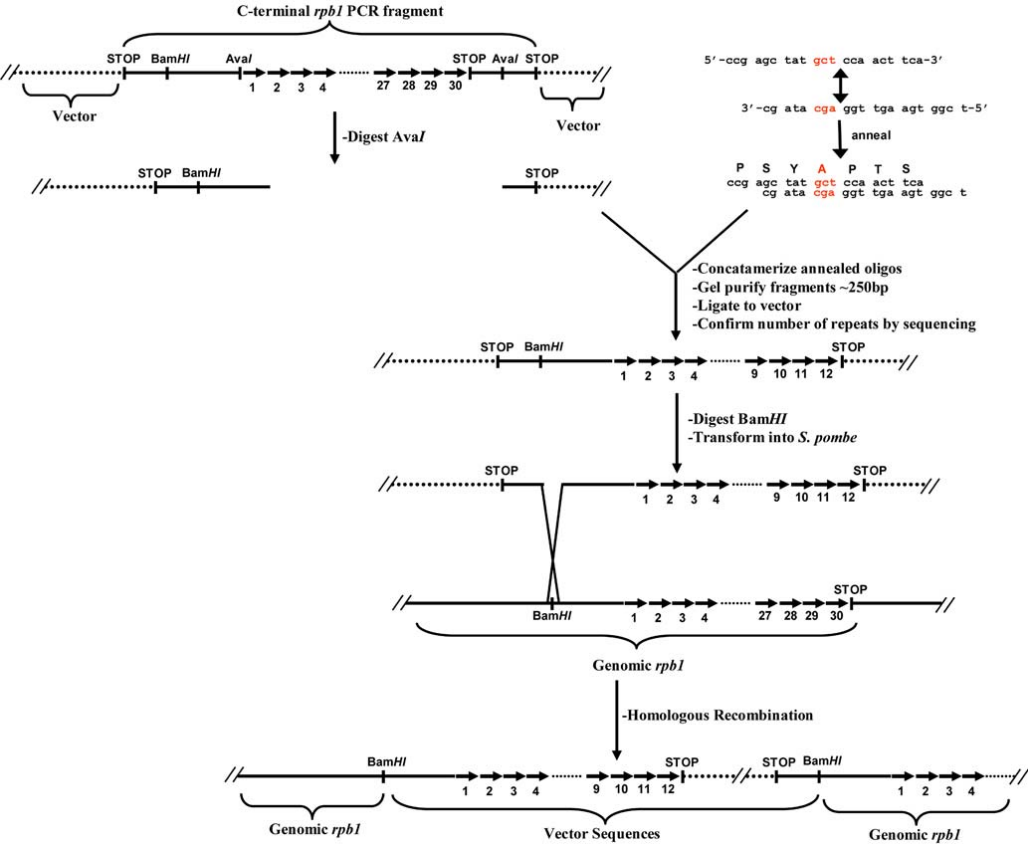
Schematic describing the creation of *rpb1-12xCTD* and *rpb1-12xS2ACTD* strains. (See Materials and Methods for details.)

Since the CTD contains approximately 30 heptad repeats, a standard site-directed mutagenesis approach to alter serine to alanine in the CTD of Rpb1p would have been impractical. Thus, we utilized a method adapted from West and Corden [32] to create two Rpb1p mutant strains through the concatamerization of DNA fragments (constituting one heptad repeat) derived from annealed oligonucleotides (Figure 5; see Materials and Methods for details). As, confirmed by DNA sequence analysis, these strains carried *rpb1* alleles encoding only 12 copies of either the wild-type heptad (YSPTSPS) or mutant heptad (YAPTSPS) sequence. Importantly, these mutant *rpb1* genes were integrated by homologous recombination and were expressed under the control of the native *rpb1* promoter. Proper integration of the constructs was confirmed by colony PCR (Figure S2). These strains will be hereafter referred to as *rpb1-12xCTD* and *rpb1-12xS2ACTD*, respectively

**Figure 6:**
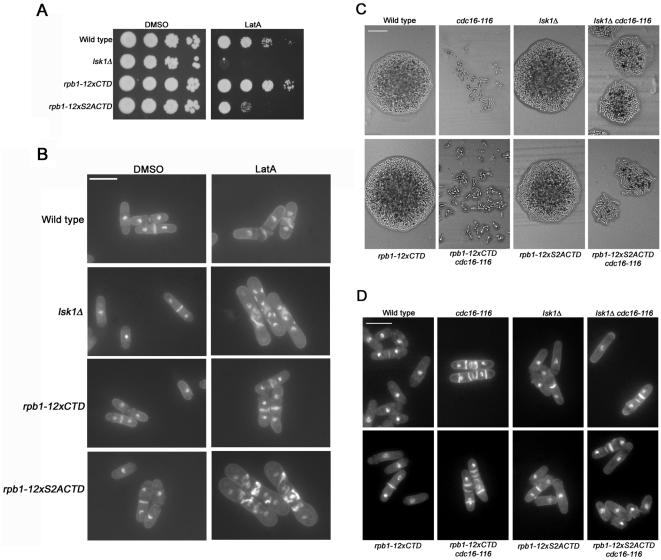
Mutation of Ser-2 to alanine in the heptad repeats of the carboxy-terminal domain of Rpb1p results in cytokinesis defects. (A) Ten-fold serial dilutions of logarithmically growing cultures were plated onto YES plates containing 0.5 µM LatA or DMSO (solvent control) and incubated at 32°C for 3 days. (B) Cells of the indicated genotype were grown to mid-log phase at 32°C and then treated with 0.3 µM LatA for 5 hours before being fixed and stained with DAPI (nuclei) and aniline blue (cell wall/septa). Bar, 10 µM. (C) Cells of the indicated genotype were freshly streaked to YES plates and incubated for 24 hours at 34°C. Bar, 50 µM. (D) Cells of the indicated genotype were grown to mid-log phase at 24°C and then shifted to 34°C for 5 hours before being fixed and stained with DAPI (nuclei) and aniline blue (cell wall/septa). Bar, 10 µM.

Haploid *rpb1-12xCTD* strains appeared morphologically normal, displayed wild-type growth rates, and a similar response to LatA as wild-type controls. In contrast, *rpb1-12xS2ACTD* strains, similar to *lsk1*Δ and *lsc1*Δ mutants, displayed a characteristic sensitivity to LatA, and an inability to complete septum formation (Figure 6, A and B; Table 1). As expected, when these strains were examined with antibodies specific for phospho-Ser-5 CTD we were able to detect lesser intensity bands with increased mobility in both *rpb1-12xCTD* and *rpb1-12xS2ACTD* strains (Figure 3, middle panel, lanes 9–12). Also as expected (since the Ser-2 residue is a critical part of the epitope recognized by the antibody against unphosphorylated CTD) [24], [25], down-shifted and lesser intensity bands could also be detected in *rpb1-12xCTD*, but not in *rpb1-12xS2ACTD* strains (Figure 3, top panel, lanes 9–12).

However, while we were unable to detect phospho-Ser-2 CTD in *rpb1-12xS2ACTD* strains as one would predict, this observation was made moot since we were also unable to detect phospho-Ser-2 CTD in *rpb1-12xCTD* strains (Figure 3). We believe this to be due to technical limitations in our ability to detect phospho-Ser-2 CTD. In contrast to the signal obtained when using the phospho-Ser-5 specific antibody (which was readily detectable), signals obtained when utilizing the phospho-Ser-2 specific antibody were consistently only slightly above background. Thus, we believe that the reduction in the number of CTD repeats (from 30 to 12) may have reduced phospho-Ser-2 levels beyond our capability of detection.

To further examine the phenotypic consequences of the *rpb1* mutations, we performed genetic crosses with *cdc16-116* and *cdc14-118* mutants. Interestingly, the *rpb1-12xS2ACTD* strain, unlike *rpb1-12xCTD*, was able to suppress the lethal multi-septate phenotype conferred by the *cdc16-116* mutation (Figure 6C and D; Table 3). In addition the *rpb1-12xS2ACTD* mutant displayed synthetically lethal genetic interactions with *ts* mutations in the SIN component *cdc14* at the semi-permissive temperature of 30°C (Figure S3, A and B, Table S1). Taken together, these results strongly suggest that the Lsk1p-Lsc1p complex promotes SIN function and thus actomyosin ring integrity by promoting Ser-2 phosphorylation of the heptad repeats found in the Rpb1p sub-unit of RNA pol II.

**Table 3:** Mean percentage of cells (±standard deviation) displaying the indicated number of septa five hours after shift from 24°C to 34°C (n = 3).

Genotype	No Septum	One Septum	Two Septa	Three Septa	>Three Septa
Wild type	89±3	11±3	0	0±0	0
*cdc16-116*	5±3	12±4	21±3	44±10	17±8
*lsk1Δ*	89±3	11±3	0	0	0
*lsk1Δ cdc16-116*	80±1	9±1	8±3	2±3	1±1
*rpb1-12xCTD*	85±2	15±2	0	0	0
*rpb1-12xCTD cdc16-116*	12±6	15±3	10±6	43±4	19±13
*rpb1-12xS2ACTD*	89±3	11±3	0	0	0
*rpb1-12xS2ACTD cdc16-116*	74±5	13±2	7±4	3±1	3±2

To further examine the effects of Ser-2 mutations, we proceeded to create an *rpb1* strain bearing twelve copies of YEPTSPS heptads in order to mimic the constitutively phosphorylated state. Interestingly, when heterozygous diploid strains bearing the *rpb1-12xS2ECTD* mutations were sporulated, tetrads gave rise to only two viable spores that were phenotypically ura^−^. When examined after 4 days growth at 32°C, the inviable progeny had germinated, but formed only microcolonies (Figure S4). Germination of spores on 1.2M sorbitol plates (which often rescues cell wall/morphogenetic defects) did not alleviate the observed lethality (data not shown). Thus, while the absence of phosphorylation on Ser-2 residues is non-essential for growth, mutations that mimic Ser-2 hyperphosphorylation, can not be tolerated in *S. pombe*.

## Discussion

Taken together these results support a model in which Lsk1p and Lsc1p form a cyclin dependent kinase complex that functions to promote cytokinesis through its role as a positive regulator of SIN signaling. The most surprising and intriguing aspect of this work is the mechanism by which the complex acts. Our phenotypic analysis of *rpb1-12xCTD* and *rpb1-12S2ACTD* strains suggests that lack of Lsk1p-Lsc1p dependent phosphorylation of Ser-2 residues located in the heptad repeats of Rpb1p is responsible for the cytokinesis phenotypes observed in the *lsk1Δ* and *lsc1Δ* mutants.

These results raise the intriguing possibility that alterations in CTD phosphorylation play a more specific and directed role in the regulation of discrete pathways than previously thought. Alternatively, the observed phenotypic effects may simply represent an indirect, and potentially random consequence of altering the core RNA pol II machinery. Two recent findings would seem to support the former interpretation.

First, work in *S. pombe* has clearly shown that alterations in CTD phosphorylation do not necessarily result in generalized effects on the overall level of transcription. In their recent work, Lee et al., [33] demonstrate that the reduction of Ser-5 phosphorylation (through mutation of the *pmh1* or *mcs6* genes) leads to the specific reduction of a sub-set of transcripts involved in the regulation of cell separation. Furthermore, the *ts pmh1-26*, and *mcs6^ts1^* mutants die with a cell separation phenotype characteristic of mutants that are unable to fully degrade the septum after cytokinesis. Thus, this specific phenotype together with the specific effect on the transcription of a sub-set of genes, showsin principle that changes in CTD phosphorylation can result in selective alterations in transcription and phenotype.

Secondly, the phosphorylation status of the CTD can be affected by environmental conditions. For example, the level of Ser-2 phosphorylation increases both upon heat shock, and during the diauxic shift in *Saccharomyces cerevisiae* [34]. In addition, Ser-2 phosphorylation of the CTD (mediated by Ctk1p) is also seen to increase upon exposure to DNA damaging agents such as hydroxyurea as well as UV irradiation. Furthermore, such alterations led to dramatic changes in the transcript levels of genes involved in the response to stress, as well as in DNA repair [22]. This responsiveness to environmental conditions, together with specific effects on transcripts required for a wild-type response to the initiating environmental insult, suggests that changes in CTD phosphorylation plays a regulatory role in the control of discrete pathways within eukaryotic cells.

While it is clear that Ser-2 phosphorylation of the CTD heptads of Rpb1p plays a role in the regulation of cytokinesis, it is still a mystery how a reduction in phospho-Ser-2 levels affects RNA pol II in such a way as to impinge upon the genes required for the successful completion of cell division. The systematic creation and analysis of CTD mutant strains altered in the composition of the CTD heptad sequence, together with expression profiling, will be a powerful tool in understanding how CTD phosphorylation can affect discrete genetic pathways, such as the SIN, within eukaryotic cells.

Lastly, it is of interest to note that budding yeast strains expressing Rpb1p/Rpo21p with serine to alanine transitions at position 2 display a lethal phenotype that is suppressed by mutations in SRB9/SSN2 [35]. Srb9p is a component of the Srb8-11 complex that forms part of the RNA pol II holoenzyme. This complex is associated with, but is genetically and biochemically distinct from the Mediator complex, and can play both positive and negative roles in transcription [36]–[39]. The Srb10p and Srb11p components of this complex are themselves a CDK/cyclin pair capable of phosphorylating both Ser2 and Ser5 residues of CTD heptads in vitro [39]. How mutations in Srb9p affect downstream target genes to relieve the lethality caused by S2A mutations is not clear. Most interestingly, however, recent work has shown that Srb9p is specifically phosphorylated by Pka1p. Furthermore, genetic data indicates that this phosphorylation modulates the activity of the Srb proteins [40]. These data further imply that the core transcriptional machinery may be modified by specific signalling pathways in order to alter the transcriptional regulation of gene sub-sets.

## Materials and Methods

### Strains, media, and growth conditions

All *S. pombe* strains used in this study were cultured in either YES or MM media as indicated [41]. All genetic crosses and general yeast techniques were performed using standard methods [42]. Latrunculin A was purchased from Molecular Probes (Eugene, OR) and stored at −20°C dissolved in DMSO at a concentration of 10 mM.

### Fluorescence Microscopy

Images were obtained using a Leica DMIRE2 microscope equipped with Uniblitz shutter and CoolSnap HQ CCD camera (Photometrics) driven by MetaMorph 6.1 software (Universal Imaging corporation). Cell staining with 4'6,-diamidino-2-phenylindole (DAPI), aniline blue, was performed as previously described [43]. Immunofluorescence was performed as previously described [43]. Rabbit anti-GFP (Molecular Probes A-6455, Eugene, OR), and mouse anti-TAT1 primary antibodies were used at 1∶800 and 1∶200 dilutions, respectively. Goat anti-rabbit IgG (MolecularProbes A-11008, Eugene OR), and Goat anti-mouse IgG (MolecularProbes A-11020, Eugene, OR) secondary antibodies, Eugene, OR) were used at a dilution of 1∶1000.

### Immunological Methods

Immuno-precipitation, immuno-blotting, and chemi-luminescent detection was performed as previously described [2] using anti-HA (12CA5, Sigma) or anti-myc antibodies (9E10, Sigma) as indicated. In experiments assessing the phosphorylation state of the Rpb1p CTD, immunoblotting was performed using primary antibodies specific to the unphosphorylated CTD (8WG16, Covance), the Ser-5 phosphorylated CTD (H14, Covance), or Ser-2 phosphorylated CTD (H5, Covance). 8WG16 and H14 antibodies were used at a 1∶5000 dilution, whereas H5 was used at a dilution of 1∶1000. Peroxidase-conjugated anti-mouse IgG (Jackson ImmunoResearch) secondary antibodies at a dilution of 1∶20000 were used against 8WG16, whereas peroxidase-conjugated anti-mouse IgM (Sigma) secondary antibodies at a dilution of 1∶5000 were used against both H14 and H5.

### Genetic Methods

To create the *rpb1-12xCTD* and *rpb1-12xS2ACTD* strains, a C-terminal fragment of the *rpb1* gene was amplified in a three step procedure. The first fragment was amplified from wild-type genomic DNA using the Expand High Fidelity PCR kit (Roche) using the primers JK76 (5’-ggg ggg gta cct gag gaa tgg gta ctt ccc aat ta-3’) and JK75 (5’-gaa gga ctc cgg ggg cat ata ag-3’). The second using the primers JK74 (5’-ctt ata tgc ccc cga gtc ctt c-3’) and JK77 (5’-ggg ggg agc tct caa ctc ggg cta aga tgg gct agt agg gga-3’). Primers JK74 and JK75 incorporated an *Ava*I site (CCCGAG) upstream of the start of the CTD sequence. Lastly, a third reaction using primers JK76, JK77, and the products of the previous two reactions as template, was performed. In this way we created a C-terminal PCR fragment of *rpb1* incorporating the *Ava*I site described above. In addition a second *Ava*I site (CTCGGG), and an in-frame STOP codon was incorporated via the JK77 primer downstream of the native *rpb1* stop codon. *Kpn*I and *Sac*I sites were also incorporated via the JK76 and JK77 primers. This PCR amplicon was subsequently cloned into the *Kpn*I and *Sac*I sites of pCDL1034 generating the pJK210-CTD plasmid.

In parallel single strand oligos of the sequence JK70 (5’-ccg agc tat agt cca act tca-3’) and JK71 (5’-tcg gtg aag ttg gac tat agc-3’); JK72 (5’-ccg agc tat gct cca act tca-3’) and JK73 (5’-tcg gtg aag ttg gag cat agc-3’); JK78 (5'-ccg agc tac gaa cca aca tca-3') and JK79 (5'-tcg gtg atg ttg gtt cgt agc-3') were synthesized. When these pairs of oligos were annealed they created sequences encoding a single heptad repeat, bearing either the wild-type sequence, or sequences encoding either a serine to alanine, or serine to glutamate transition at the S2 position. Resultant dsDNA fragments were also designed to bear ssDNA overhangs that allowed the uni-directional concatamerization of the fragments. Oligos JK71 and JK72, JK72 and JK73, as well as JK78 and JK79 were separately annealed by combining the individual pairs of oligos at a concentration of 50 µM in annealing buffer (100 mM Tris pH 8.0, 10 mM EDTA, 500 mM NaCl). The reaction was subsequently incubated at 95°C for 5 minutes, and the reaction cooled to 25°C at a rate of 1°C per minute in a thermocycler.

Two micrograms of annealed DNA fragments were subsequently phosphorylated in 10×ligase buffer (New England Biolabs) with T4 polynucleotide kinase (Promega) for 30 minutes at 37°C. Reactions were subsequently heat inactivated at 65°C for 20 minutes. Due to the design of the ssDNA overhangs, these annealed, phosphorylated DNA fragments could then be directionally concatamerized in a standard ligation reaction. Products of these ligations were run on a 2% agarose gel and fragments in the size range of ∼200–400 bp were gel purified (Qiagen). The gel purified products were then used in a second ligation reaction, with *Ava*I digested pJK210-CTD plasmid. Again due to the design of the ssDNA overhangs this allowed for the directional cloning of the concatamerized DNA fragments. Resultant plasmids were sequenced to determine the number of heptad repeats present. Plasmids bearing 12 copies of the WT, S2A, or S2E heptads were subsequently digested with *Bam*HI and transformed into a diploid strain to obtain integrants. The desired integration was confirmed by colony PCR, using the primers JK82 (5’-act ggc gca ttt gat att ta-3’) and MOH1207 (5’-aat taa ccc tca cta aag gg-3’). Haploid strains were phenotypically analyzed from sporulated diploids.

To create the *lsc1* deletion strain two separate DNA fragments, one upstream and one downstream of the *lsc1* open reading frame were PCR amplified from wild-type genomic DNA using the primers JK29 (5’-ggg ggg gta ccg ctt tca gga aat gtt tgg cgt-3’), JK30 (5’-ggg ggc tcg agg cct ggc ttt acc tgc aag gtg-3’) and JK31: 5’-ggg ggt cta gaa agc agc tga cct aaa ccc tgc-3’), JK32 (5’-ggg ggg agc tct tcc cac cat tag cgt gat tgc-3’). The JK29-JK31 generated fragment was subsequently cloned (using *Kpn*I and *Xho*I sites) into the pCDL126 plasmid upstream of the *Hin*dIII site (this plasmid contains a *Hin*dIII fragment bearing the *ura4* selectable marker). The JK31-JK32 generated fragment was then subsequently cloned downstream of the *Hin*dIII fragment using *Xba*I and *Sac*I sites. When digested with *Kpn*I and *Sac*I this liberated a linear fragment containing *lsc1* upstream sequence, the *ura4* fragment, and *lsc1* downstream sequences. This fragment was subsequently transformed into *S. pombe* to obtain integrants. Correct integration at the *lsc1* locus was confirmed by colony PCR.

To create the Lsc1-GFP integrant strain a C-terminal fragment of the *lsc1* gene was PCR amplified from genomic DNA using primers JK53 (5’-ggg ggg aat tct gac atg tac ttt caa tac gaa tgt-3’) and JK54 (5’-gct acc cgg gaa ccg tac ctt tat ttc tcc t-3’) using the Expand high-fidelity PCR system (Roche) and cloned into the unique Eco*RI* and Sma*I* sites of the pJK210-GFP vector. Plasmids were linearized by digestion with *Xba*I and transformed into *S. pombe* to obtain integrants. The correct integration at the *lsc1* locus was confirmed by colony PCR.

To create the Fcp1-GFP integrant a C-terminal fragment of the *fcp1* gene was PCR amplified from genomic DNA using primers JK80: 5’-ggggggaattctgagctgtatctatgggaaatattaa-3’ and JK81: 5’-gggggcccgggagctgtatctttggacaattca-3’ using the Expand high-fidelity PCR system (Roche) and cloned into the unique Eco*RI* and Sma*I* sites of the pJK210-GFP vector. Plasmids were linearized by digestion with Bgl*II* and transformed into *S. pombe* to obtain integrants. The correct integration at the *fcp1* locus was confirmed by colony PCR.

To create the *lsk1-K306R* HA tagged mutant, A N-terminal fragment of the *lsk1* gene was PCR amplified using primer JK6 (5’-ggg ggg aat tcg aac agc gga aaa gaa cat tt-3’) and a reverse primer incorporating the K306R mutation, JK47 (5’-cct tat ccg tcg taa agc tac-3’). Similarly, a C-terminal fragment of the *lsk1* gene was PCR amplified with a JK7 (5’-ggg ggc ccg ggt ctt tta gat ttt cgt ttt tta c- -3’) and a forward primer incorporating the K306R mutation, JK48 (5’-gta gct tta cga cgg ata agg -3’). A full length product containing the desired mutations was then PCR amplified using JK6, JK7, and the products of the first two reactions as template. The full length product was then cloned into the *Eco*RI and *Sma*I sites of the pCDL898 vector. The vector was linearized with *Xba*I and transformed into *S. pombe* to obtain integrants. The correct integration at the *lsk1* locus was confirmed by colony PCR. A similar strategy was used to create the *lsk1-K306R* GFP tagged mutant, only the full length product was cloned into the *Eco*RI and *Sma*I sites of the pJK210-GFP vector.

The pREP1-*lsk1CFP* vector was created by PCR amplifying the *lsk1* gene using primers JK18 (5’-ggg gga tta ata tgt cat act-3’)and JK7 (5’-ggg ggc ccg ggt ctt tta gat ttt cgt ttt tta c-3’) and cloning it, in a single reaction with a *Sma*I*-Bam*HI fragment containing the CFP gene into the *Nde*1 and *Bam*HI sites of the pREP1 vector. The pREP1-*lsc1GFP* vector was created by PCR amplifying the *lsc1* gene using primers JK55 (5’-ggg ggc ata tgg cag aaa atg aga atc at-3’) and JK56 (5’-ggg ggc ccg ggt taa acc gta cct tta ttt ctc-3’) and cloning it, in a single reaction along with a *Nde*I fragment containing the GFP gene, into the *Nde*I and *Sma*I sites of the pREP1 vector. To create the pREP2-*lsc1GFP* vector, a *Pst*I *Sac*I fragment containing the *nmt1* promoter, *lsc1* gene, and *nmt1* terminator sequences, was cloned into the *Pst*I and *Sac*I sites of the pREP2 vector.

To create the Lsk1-HA integrant strain a C-terminal fragment of the *lsk1* gene was PCR amplified from genomic DNA using primers JK6 (5’-ggg ggg aat tcg aac agc gga aaa gaa cat tt-3’) and JK7 (5’-ggg ggc ccg ggt ctt tta gat ttt cgt ttt tta c-3’) using the Expand high-fidelity PCR system (Roche) and cloned into the unique *Eco*RI and *Sma*I sites of the pCDL898 vector. Plasmids were linearized by digestion with *Xba*I and transformed into *S. pombe* to obtain integrants. The correct integration at the *lsk1* locus was confirmed by colony PCR.

To create the Lsc1-myc integrant strain a C-terminal fragment of the *lsc1* gene was PCR amplified from genomic DNA using primers JK62 (5’-ggg gg ggt acc tga cat gta ctt tca ata cga atg t-3’) and JK54 (5’-gct acc cgg gaa ccg tac ctt tat ttc tcc t-3’) using the Expand high-fidelity PCR system (Roche) and cloned into the unique *Kpn*I and *Sma*I sites of the pCDL592 vector. Plasmids were linearized by digestion with *Xba*I and transformed into *S. pombe* to obtain integrants. The correct integration at the *lsc1* locus was confirmed by colony PCR.

## Supporting Information

Figure S1 Lsk1p and Fcp1p co-localize to the nucleus. Strains expressing Fcp1-GFP (integrated at its normal genomic locus, and under the control of its native promoter) as well as plasmid borne Lsk1-CFP (under the control of the thiamine repressible *nmt1* promoter) were grown for 12 hours in minimal media in the absence of thiamine, and then imaged in the CFP and YFP channels, respectively. Bar, 3 microns.
(0.91 MB TIF)

Figure S2 Colony PCR reactions confirming the integration of *rpb1-12xCTD* and *rpb1-12xS2ACTD* constructs. Cells of the indicated genotype were freshly streaked to YES and used as template in a colony PCR assay. PCR reactions were subsequently analyzed by agarose gel electrophoresis. As a control, a strain bearing 16 heptad repeats (*rpb1-16xS2ACTD*) was included in the analysis. PCR amplicons obtained from this strain displayed a band-shift consistent with the presence of four extra heptad repeats relative to *rpb1-12xCTD* and *rpb1-12xS2ACTD* strains.
(0.23 MB TIF)

Figure S3 *rpb1-12xS2ACTD cdc14-118* double mutants are inviable at 30°C due to cytokinesis failure. (A) Cells of the indicated genotype were freshly streaked to YES plates and incubated for 24 hours at 30°C. Bar, 50 microns. (B) Cells of the indicated genotype were grown to mid-log phase at 24°C and then shifted to 30°C for 5 hours before being fixed and stained with DAPI (nuclei) and aniline blue (cell wall/septa). Bar, 10 microns.
(1.52 MB TIF)

Figure S4 Mutation of Ser-2 to Glutamate in the heptad repeats of the carboxy-terminal domain of Rpb1p is lethal in *S. pombe.* (A) Heterozygous diploid strains bearing the *rpb1-12xS2ECTD* mutation were sporulated. The spores of individual asci were then separated and grown on YES plates for 3 days at 32°C. Three individual tetrads displaying the observed 2:2 segregation of viable to inviable progeny are shown. (B) Four separate examples of the colony morphology observed when inviable spores were examined by brightfield microscopy. Bar, 20 microns.
(0.27 MB TIF)

Table S1 Mean percentage of cells (+/− standard deviation) displaying the indicated number of nuclei five hours after shift from 24°C to 30°C (n = 3).
(0.04 MB DOC)
