# Supplementary figures and images for: A Cyclin-Dependent Kinase that Promotes Cytokinesis through Modulating Phosphorylation of the Carboxy Terminal Domain of the RNA Pol II Rpb1p Sub-Unit
Source: PLoS One. 2007 May 9;2(5):e433. doi: 10.1371/journal.pone.0000433 (PMC1855991; doi:10.1371/journal.pone.0000433)

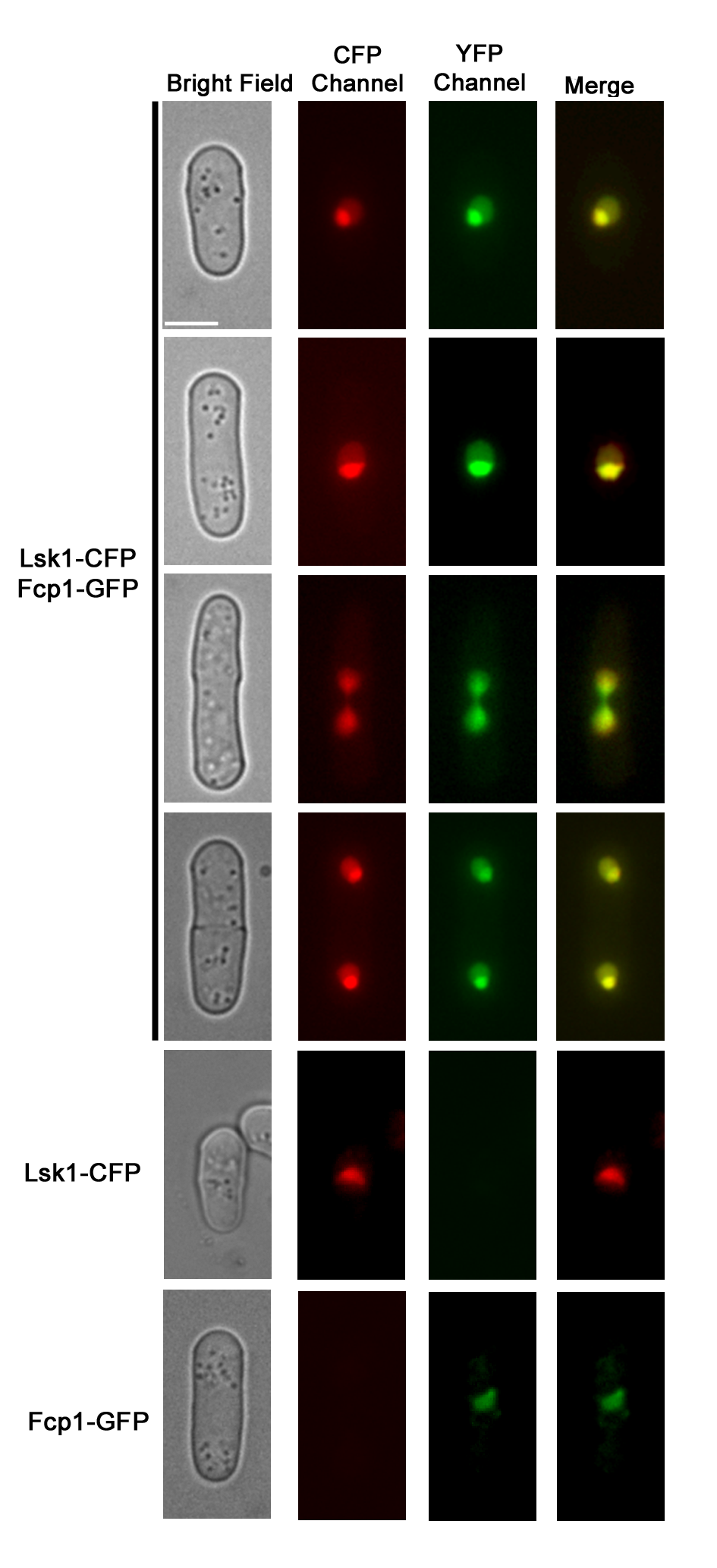

Supplement: Figure S1 — Lsk1p and Fcp1p co-localize to the nucleus. Strains expressing Fcp1-GFP (integrated at its normal genomic locus, and under the control of its native promoter) as well as plasmid borne Lsk1-CFP (under the control of the thiamine repressible nmt1 promoter) were grown for 12 hours in minimal media in the absence of thiamine, and then imaged in the CFP and YFP channels, respectively. Bar, 3 microns. (0.91 MB TIF) [file pone.0000433.s001.tif]

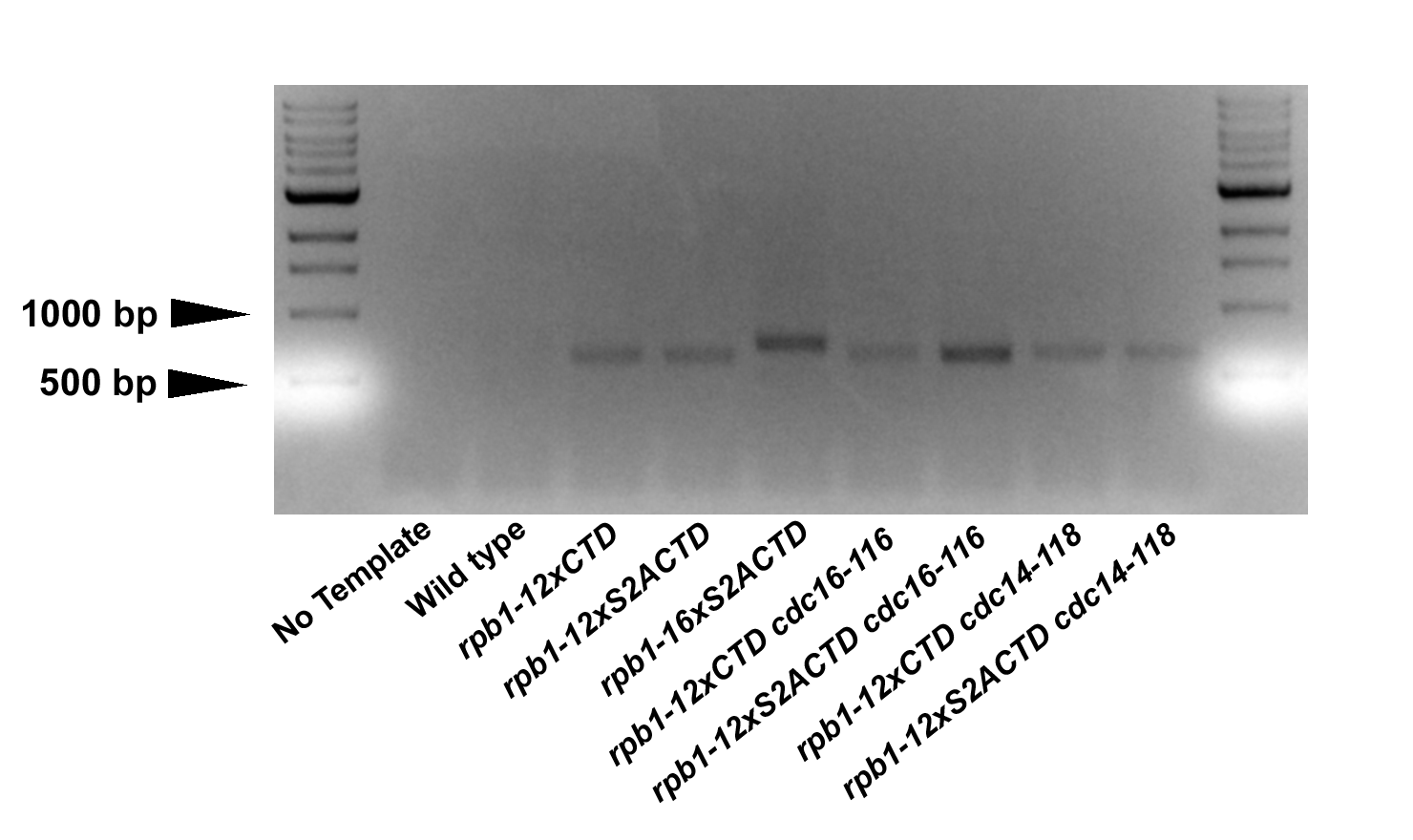

Supplement: Figure S2 — Colony PCR reactions confirming the integration of rpb1-12xCTD and rpb1-12xS2ACTD constructs. Cells of the indicated genotype were freshly streaked to YES and used as template in a colony PCR assay. PCR reactions were subsequently analyzed by agarose gel electrophoresis. As a control, a strain bearing 16 heptad repeats (rpb1-16xS2ACTD) was included in the analysis. PCR amplicons obtained from this strain displayed a band-shift consistent with the presence of four extra heptad repeats relative to rpb1-12xCTD and rpb1-12xS2ACTD strains. (0.23 MB TIF) [file pone.0000433.s002.tif]

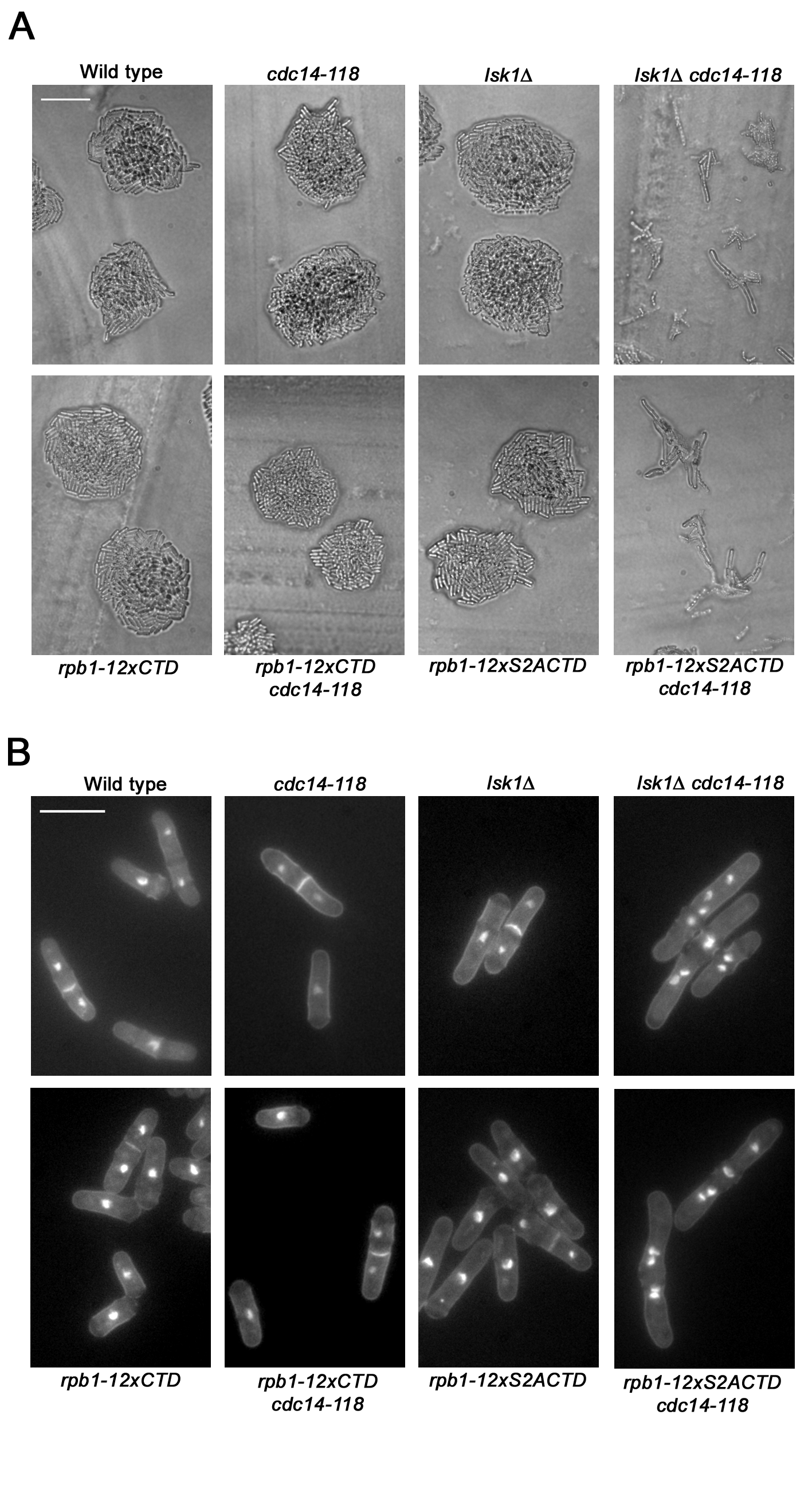

Supplement: Figure S3 — rpb1-12xS2ACTD cdc14-118 double mutants are inviable at 30°C due to cytokinesis failure. (A) Cells of the indicated genotype were freshly streaked to YES plates and incubated for 24 hours at 30°C. Bar, 50 microns. (B) Cells of the indicated genotype were grown to mid-log phase at 24°C and then shifted to 30°C for 5 hours before being fixed and stained with DAPI (nuclei) and aniline blue (cell wall/septa). Bar, 10 microns. (1.52 MB TIF) [file pone.0000433.s003.tif]

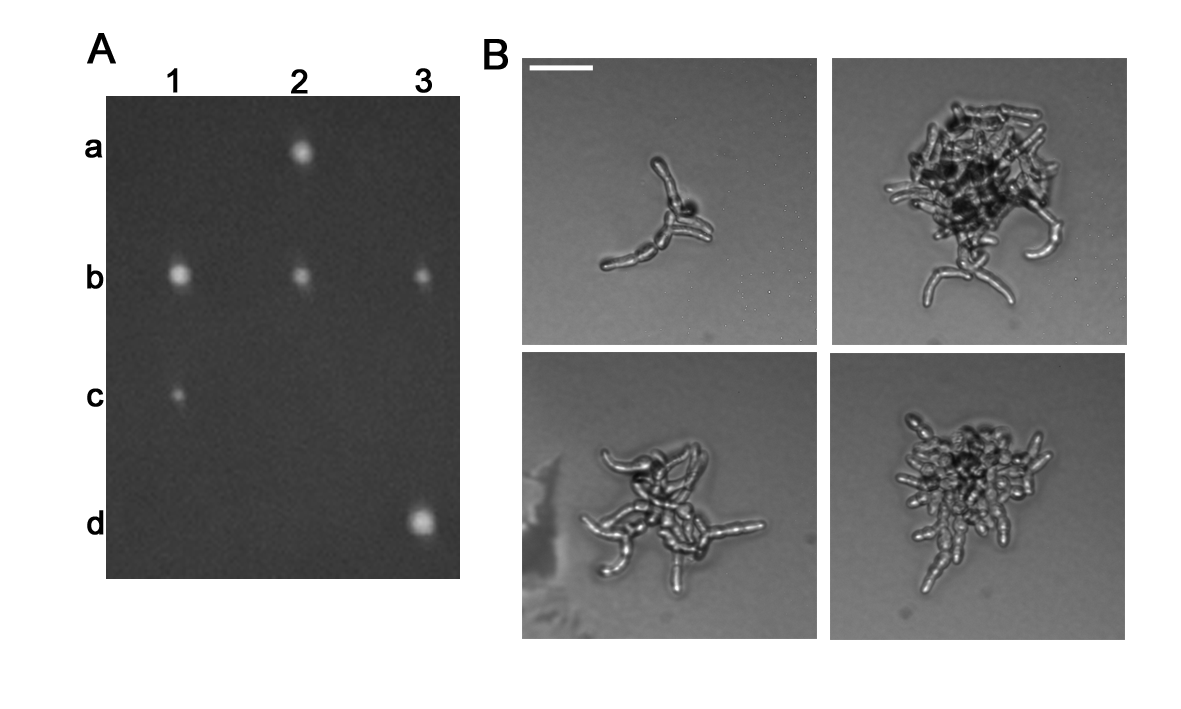

Supplement: Figure S4 — Mutation of Ser-2 to Glutamate in the heptad repeats of the carboxy-terminal domain of Rpb1p is lethal in S. pombe. (A) Heterozygous diploid strains bearing the rpb1-12xS2ECTD mutation were sporulated. The spores of individual asci were then separated and grown on YES plates for 3 days at 32°C. Three individual tetrads displaying the observed 2:2 segregation of viable to inviable progeny are shown. (B) Four separate examples of the colony morphology observed when inviable spores were examined by brightfield microscopy. Bar, 20 microns. (0.27 MB TIF) [file pone.0000433.s004.tif]
