# Supplementary material for: A Cyclin-Dependent Kinase that Promotes Cytokinesis through Modulating Phosphorylation of the Carboxy Terminal Domain of the RNA Pol II Rpb1p Sub-Unit
Source: PLoS One. 2007 May 9;2(5):e433. doi: 10.1371/journal.pone.0000433 (PMC1855991; doi:10.1371/journal.pone.0000433)
Supplement: Table S1 — Mean percentage of cells (+/− standard deviation) displaying the indicated number of nuclei five hours after shift from 24°C to 30°C (n = 3). (0.04 MB DOC) [file pone.0000433.s005.doc]

**Table S1.** Mean percentage of cells (± standard deviation) displaying the indicated number of nuclei five hours after shift from 24°C to 30°C (n=3).

| **Genotype** | **Uni-nucleate** | **Bi-nucleate** | **Tetra-nucleate** | **> 4 Nuclei** |
| --- | --- | --- | --- | --- |
| Wild type | 89 ± 3 | 11 ± 3 | 0 | 0 |
| *cdc14-118* | 90 ± 4 | 9 ± 3 | 1 ± 1 | 0 |
| *lsk1Δ* | 88 ± 3 | 12 ± 3 | 0 | 0 |
| *lsk1Δ cdc14-118* | 14 ± 2 | 27 ± 1 | 58 ± 3 | 3 ± 1 |
| *rpb1-12xCTD* | 90 ± 1 | 10 ± 1 | 0 | 0 |
| *rpb1-12xCTD cdc14-118* | 87 ± 4 | 11 ± 2 | 2 ± 3 | 0 |
| *rpb1-12xS2ACTD* | 89 ± 3 | 12 ± 3 | 0 | 0 |
| *rpb1-12xS2ACTD*  *cdc14-118* | 14 ± 6 | 25 ± 8 | 59 ± 5 | 2 ± 1 |
